# Editorial: Neglected nuances in gestational diabetes

**DOI:** 10.3389/fcdhc.2025.1706918

**Published:** 2025-10-20

**Authors:** Jyoti S. Mathad, Chittaranjan S. Yajnik

**Affiliations:** ^1^ Center for Global Health, Weill Cornell Medicine, Cornell University, New York, NY, United States; ^2^ Kamalnayan Bajaj Diabetology Research Centre, King Edward Memorial Hospital Research Centre, Pune, India

**Keywords:** gestational diabetes, pregnancy, pathogenesis, mixed nutrient, periconceptional programming, LMIC

The purpose of this Research Topic is to reconsider our ideas about gestational diabetes (GDM). There is no universal agreement on the definition of GDM (1). There is no consensus on the need for a screening test, the dose of the glucose load, the timing of the glucose tolerance test, the number and timings of post-glucose load measurements, or the cutoff points that define glucose intolerance. There is also no consensus on which adverse outcomes are the most important. These inconsistencies result in diagnostic practices that vary widely within and between countries. We have been too “glucocentric” in our approach. We have also overlooked the life course effects in the mother and offspring. A critical re-evaluation is warranted, looking at mother’s pre-pregnancy metabolism, to improve long-term outcomes in the offspring. To understand how we got here, let’s start at the beginning.

## The origins of gestational diabetes

The original cutoffs for pregnancy hyperglycemia described in 1964 by O’Sullivan and Mahan were based on the mother’s future diabetes risk (2). Subsequently, the National Diabetes Data Group (NDDG), sponsored by the US National Institutes of Health, revised these cutoffs to focus on short-term outcomes such as fetal overgrowth (3). The NDDG’s definition of GDM was “a condition of women in whom glucose intolerance develops or is discovered during pregnancy.” In 1980, Norbert Frienkel re-introduced the concept of long-term consequences both for the mother and the offspring (4). His ‘mixed-nutrient teratogenesis’ hypothesis suggested that prenatal exposure to increased levels of mixed nutrients such as lipids, glucose, and amino acids promotes fetal overgrowth and increases risk of future obesity and diabetes in the offspring. Studies on Akimel O'odham people confirmed that maternal diabetes perpetuates the vicious intergenerational cycle of diabetes and obesity. Despite these formative studies, an increasing amount of research has focused solely on glucose management and on short-term GDM outcomes (5–7).

## The evolution of the definition of GDM

Our understanding of GDM gained some consensus in 2008 with the multinational Hyperglycemia and Adverse Pregnancy Outcomes (HAPO) study, an observational longitudinal study of 23,316 pregnant women. HAPO described a continuous, graded association of fasting and post-load glycemia with adverse short-term outcomes, such as macrosomia, C-section rate, elevated cord blood C-peptide, and neonatal hypoglycemia (5). The International Association of Diabetes in Pregnancy Study Group (IADPSG), therefore, appointed a committee which defined GDM by somewhat arbitrary consensual cutoffs that have now become the most widely used worldwide (8). Because the diagnosis is based on glucose cutoffs, the clinical approach to GDM is almost entirely glucocentric. Only occasionally has attention been given to management of comorbidities such as obesity, smoking history, or chronic hypertension or consideration of maternal age, gravidity, and fetal sex, as highlighted in Ye et al. (9) Interestingly, long-term risks to the infant disappeared from all GDM criteria to date, along with long-term maternal risks.

## Is GDM truly a disorder of pregnancy?

The prevalent thinking is that GDM appears during pregnancy and remits with delivery due to placenta-related changes. However, evidence suggests that metabolic changes predate pregnancy. In 1999, Catalano, et al. showed that women who were diagnosed with GDM already had higher glycemia and insulin resistance before pregnancy (10). A number of clinical studies and follow-ups in long-term cohorts have confirmed higher glycemia and other risk factors for diabetes (obesity or dyslipidemias) years before pregnancy (11, 12). A recent report traces maternal pregnancy glycemia to early childhood and serially into puberty and young adult age, and even her mother’s glycemia when pregnant with the index daughter (13). This suggests that their insulin resistance was not a *de novo* gestational feature. Many cases of GDM represent detection of a chronic, often progressive, loss of B cell compensation that neither develops during nor depends on pregnancy to manifest (14). In other words, not all GDM begins with gestation.

Maternal pre-pregnancy obesity, dyslipidemias, and the glucose-insulin metabolism are major determinants of both short- and long-term adverse pregnancy outcomes (13, 14). For example, Pettit and Jovanovic showed that a woman’s own birthweight was significantly associated with risk of developing hyperglycemia in pregnancy, suggesting that metabolic programming happens very early in life (15). The crucial period for fetal programming is pre-and periconceptional (before implantation) and is influenced by the mother’s pre-pregnancy characteristics (16, 17). These considerations are completely missed in our current thinking of diagnosing and managing GDM. Even the recent interest in early GDM (diagnosis before 20 weeks) may be too late to prevent fetal programming of diabesity (18). This could explain why intensive glycemic management of women with GDM has not prevented obesity and diabetes in the offspring (19, 20). There is a need to set up studies to normalize preconceptional metabolism and find its effect on short- and long-term outcomes for the offspring.

## Mechanistic theories on GDM-related adverse outcomes

There are two major theories describing how GDM-related short- and long-term adverse outcomes develop: mixed-nutrient teratogenesis and periconceptional programming.

- The mixed-nutrient teratogenesis theory describes how increased maternal insulin resistance leads to the shuttling of multiple nutrients—glucose, lipids, and amino acids—to the fetus, which promotes fetal overgrowth and future diabesity risk (4). Of these nutrients, we focus only on glucose because it is easily measured in clinical practice and has treatments approved for pregnant women. Other nutrients are completely ignored. Lipids, for example, are major mediators of fetal overgrowth. Most obstetricians do not test lipids during pregnancy (21–23), likely because there are few pharmacological treatment options for hyperlipidemia in pregnancy. Similarly, multiple amino acids are associated with hyperglycemia in pregnancy but are also not routinely considered (24). Our group has shown that, in a predominantly vegetarian Indian cohort, maternal folate was positively related to fetal growth and subsequent adiposity, while low B12-high folate status in the mother predicted insulin resistance in young offspring (25). Current guidelines recommend only iron and folic acid during pregnancy. Excess folate has been associated with other adverse outcomes including increased prevalence of GDM (26–28).
- Periconceptional programming refers to the effect of the intrauterine environment on traits that appear later in life. Hales and Barker introduced this concept in 1991 when they noted that lower birthweight was associated with higher risk of T2D in adulthood (29, 30). Later on, this association was shown to be U-shaped: larger birthweight babies also had a higher risk of future T2D and obesity (31). Similarly, a U-shaped association was shown between birthweight of the mother and her risk of GDM: birthweights at either extreme had an increased risk (15). The mechanisms involved in fetal programming and teratogenesis are largely epigenetic and therefore modifiable (32–34). The epigenetic mechanism most studied is DNA methylation, which influences gene expression. Fetal DNA methylation is affected by various pre-pregnancy maternal factors (nutrition, metabolism, stress, etc.) (35, 36). Cord blood DNA methylation also predicts obesity (37) and glucose-insulin metabolism (38). These epigenetic changes promote the vicious cycle of intergenerational diabesity. Breaking this cycle will require targeting maternal nutrition and metabolism *prior to* pregnancy to influence pre- and periconceptional events (16, 17).

## *Primum non nocere* (“First do no harm”)

Guidelines that solely focus on lowering glucose during pregnancy could result in harm for certain populations. For example, 20% of pregnant Indian mothers are underweight and 17% of infants are born at low birthweight (<2.5 kg) (39). Average weight gain in pregnancy is significantly lower than international standards (40). In underweight and normal weight women, a substantial proportion of GDM (>75%) is diagnosed through mildly elevated fasting plasma glucose alone (41). Non-overweight women are at risk of low birthweight deliveries. Aggressive management of mild glucose intolerance, then, can worsen fetal growth restriction and result in heightened risk of future diabesity for the infants (42). By imposing guidelines derived from the HAPO study onto chronically undernourished populations with poor fetal growth and high prevalence of low birthweight, we may be promoting further fetal growth restriction. Primum non nocere.

Mild glucose intolerance may actually be nature’s way of promoting fetal growth and counteracting maternal undernutrition. An unusual physiologic situation highlights this point beautifully. Hattersley et al. described a glucokinase mutation that causes elevated fasting glucose in the mother, lower insulin secretion in the fetus, and consequently a lower birthweight (34). In this condition (estimated 1 in 1000 people), strict maternal glycemic control could worsen fetal growth restriction.

## Synthesis and future directions

In this Research Topic focusing on ‘Neglected Nuances in Gestational Diabetes’, we present five articles that highlight the heterogenous aspects of GDM diagnosis, treatment, and prevention that are not commonly considered in clinical practice. The first article by Gitlin et al. broadens our understanding of risk factors for GDM. In their systematic review, 7% of non-overweight pregnant women have GDM globally, with 12% in Asia. SGA infants are highly prevalent among these women. Wadivkar and Hawkins challenge management algorithms in their perspective “Is gestational diabetes mellitus in lean women a distinct entity warranting a modified management approach?” They propose that standard GDM management guidelines may in fact cause harm to non-overweight women and their infants.

In the next article, Mullins et al. focus on the effect of fetal sex on GDM diagnosis, insulin dynamics, and pregnancy outcomes. They present evidence that carrying a male fetus may be associated with a decrease in the hormones needed for maternal beta cell proliferation, thereby reducing insulin secretion, fetal nutrition, and fetal growth. Fetal sex represents another factor that may be important when deciding whether and how to treat hyperglycemia in pregnancy.

Finally, Dias et al. and Liew et al. explore novel ways to prevent long-term maternal diabetes and diabetes-related health risks. Dias et al. use experience-based co-design methodology with First Nations communities to identify priority areas to reduce diabetes risks before, during, and after pregnancy. Liew et al. design and test a holistic intervention to change lifestyle behaviors among high-risk Asian women. The intervention addresses factors such as sleep and stress, which are under-appreciated risk factors for metabolic disease, in addition to the classic risk factors.

Together, these studies highlight the fact that GDM is not a ‘one-size-fits-all’ condition nor a condition that is limited to pregnancy. They call attention to the ‘subtypes’ of women with GDM that could be harmed by standard GDM treatment. GDM pathophysiology must be understood in the context of how it develops throughout a woman’s life rather than as a temporary condition that should be managed only during the pregnancy period. Only then can we mitigate the vicious intergenerational cycle of diabetes.
